# Development, characterization, and cross‐amplification of 17 microsatellite markers for *Filipendula vulgaris*

**DOI:** 10.1002/aps3.11307

**Published:** 2019-12-08

**Authors:** Dijana Čortan, Karol Krak, Petr Vít, Bohumil Mandák

**Affiliations:** ^1^ Faculty of Environmental Sciences Czech University of Life Sciences Prague Kamýcká 129 Praha 6–Suchdol CZ‐165 00 Czech Republic; ^2^ The Czech Academy of Sciences Institute of Botany Zámek 1 Průhonice CZ‐252 43 Czech Republic

**Keywords:** cross‐amplification, *Filipendula vulgaris*, microsatellites, perennial herb, Rosaceae

## Abstract

**Premise:**

Polymorphic microsatellite markers were developed as a tool for genetic investigations of *Filipendula vulgaris* (Rosaceae) and related species.

**Methods and Results:**

Seventeen new polymorphic microsatellite markers were developed for *F. vulgaris* using the Illumina MiSeq platform. Polymorphism of the 17 loci was tested in three populations. We identified a total of 203 alleles, ranging from four to 19 per locus, with levels of observed and expected heterozygosity ranging from 0.267 to 1.000 and 0.461 to 0.899, respectively. Primers were also tested for cross‐amplification in three related species. Seven loci successfully cross‐amplified in *F. camtschatica* and *F. ulmaria*, whereas we detected positive cross‐amplification in only three loci in *Geum urbanum*.

**Conclusions:**

The newly developed microsatellite primers will serve as useful genetic tools for further population genetic studies on *F. vulgaris* and related species.

The genus *Filipendula* Mill. (Rosaceae) contains 15 species of perennial herbaceous flowering plants native to temperate regions in the Northern Hemisphere (Schanzer, 1994). This genus is most diverse in eastern Asia, with only two species native to Europe and North America (Schanzer, 2016). The native European species belong to sect. *Filipendula* and are represented by *F* *. ulmaria* (L.) Maxim. and *F. vulgaris* Moench (syn. *F. hexapetala* Gilib.) (Ball, 1968). Whereas *F. ulmaria* is confined to wet habitats, the focal species *F. vulgaris* occurs in dry steppe‐like habitats. Morphologically, *F. vulgaris* is unique within *Filipendula* in having tuberous roots and strongly dissected leaves. The geographic distribution of *F. vulgaris* covers Europe, central Asia, and northwestern Africa (Meusel et al., 1965). It occurs in dry, non‐acidic grasslands in Europe (Ball, 1968) and in continental Eurasian steppes. The species is adapted to drought‐prone soils and is not sensitive to frost and low temperatures. Flowers, leaves, and underground organs are used as medicinal raw materials because they are rich in tannins and polyphenolic acids (Bączek et al., 2012).

*Filipendula vulgaris* is a perennial diploid species (2*n* = 2*x* = 14; Schanzer, 1994 and references therein). It is described as a self‐compatible but predominantly outcrossing plant with low genetic differentiation both within and among populations (Weidema et al., 2000). To date, only isozymes have been used to assess genetic variation in this species (Weidema et al., 2000). Our study reports the development and characterization of 17 novel microsatellite loci for *F. vulgaris*. In addition, these loci were cross‐amplified in three related Rosaceae species: *Filipendula ulmaria*,* F. camtschatica* (Pall.) Maxim., and *Geum urbanum* L. The microsatellite markers developed here will be used to assess genetic diversity in investigations of population genetic structures, mating systems, and phylogeographic patterns of these species.

## Methods and results

### Microsatellite development

Total genomic DNA of *F. vulgaris* was extracted from 20–25 mg of silica gel–dried leaf tissue using the DNeasy Plant Mini Kit (QIAGEN, Hilden, Germany), following the manufacturer's instructions. A DNA library was prepared with a NEBNext Ultra II DNA Library Prep Kit for Illumina (New England Biolabs, Ipswich, Massachusetts, USA) as described in Belyayev et al. (2019), and 2 × 300‐bp paired‐end sequencing was carried out on an Illumina MiSeq instrument using the services of Macrogen (Seoul, South Korea). The library was sequenced in one run together with nine other libraries. Sequencing resulted in 1,327,940 raw reads (National Center for Biotechnology Information [NCBI] Sequence Read Archive no. SRR10028579). Paired‐end reads were trimmed using Trimmomatic 0.36 (Bolger et al., 2014) with the following settings: ILLUMINACLIP: adapters_used.fa: 2:30:10; LEADING: 20; TRAILING: 20; SLIDINGWINDOW: 4:20; MINLEN: 48. Chloroplast reads of *F. vulgaris* were then removed by mapping the trimmed reads to the complete chloroplast sequence of *Rosa roxburghii* Tratt. (GenBank accession no. KX768420) using Bowtie 2 aligner with default settings (Langmead and Salzberg, 2012). Sequences containing microsatellite motifs were identified using SSR_pipeline (Miller et al., 2013), and only perfect motifs (di‐, tri‐, and tetranucleotide repeats of minimum length of 14, 18, and 20 bp, respectively) were further processed. Primers were designed using Primer3 (Untergasser et al., 2012), as integrated in MSATCOMMANDER version 0.8.2 (Faircloth, 2008).

### Biological validation

A total of 65 primer pairs containing perfect di‐, tri‐, and tetranucleotide repeats and different amplicon lengths (100–400‐bp intervals) were randomly selected and tested for amplification in seven individuals of *F. vulgaris* (Appendix 1). PCRs were carried out in 10‐μL reaction volumes containing 10 ng of genomic DNA (0.05 μM of forward and 0.2 μM of reverse primer, 1× concentrated QIAGEN Multiplex PCR Master Mix). We further added 0.2 μM of fluorescently labeled (FAM, NED, VIC, or PET) M13 primer to facilitate labeling of the resulting PCR product as described in Schuelke (2000). Reactions were carried out under the following conditions: an initial denaturation step at 95°C for 15 min, followed by 25 cycles of denaturation at 95°C for 30 s, annealing at 55°C for 30 s, and extension at 72°C for 2 min. This was followed by 10 cycles of denaturation at 95°C for 30 s, annealing at 50°C for 30 s, extension at 72°C for 2 min, and final extension at 72°C for 10 min. After verifying amplification by electrophoresis in 2% agarose gel, a total of 25 primers were selected as successfully amplified in all seven individuals and these were used for the initial polymorphism tests. A volume of 1 μL of PCR products was added to a mix of 12.0 μL Hi‐Di Formamide (Applied Biosystems, Waltham, Massachusetts, USA) and 0.2 μL GeneTrace LIZ 500 Size Standard (Carolina BioSystems, Ořech, Czech Republic) for fragment analysis on the Applied Biosystems 3500 genetic analyzer. Next, fragment length analyses and scoring were carried out with GeneMarker version 2.7.4 (SoftGenetics, State College, Pennsylvania, USA). Finally, 17 polymorphic markers with easily scorable peaks were selected for further analysis on three *F. vulgaris* populations (Table 1, Appendix 1).

**Table 1:** Characteristics of 17 polymorphic nuclear microsatellite loci developed for *Filipendula vulgaris*.^a^

Locus	Primer sequences (5′–3′)	Repeat motif	Allele size range (bp)	Fluorescent dye	GenBank accession no.
FV_di_02	F: *GCATCAACACAACAACCTCTC	(AC)_13_	177–191	FAM	MN259475
	R: ACGCCGAGACTAGAGTTTCC				
FV_di_10	F: *AAGCCACACGAAACCCAAAG	(AG)_11_	250–270	VIC	MN259479
	R: AACGGATGGACTACGCCTTC				
FV_tri_24	F: *CCATGTTAAGCCTAATCCCAC	(AAT)_15_	178–217	PET	MN259486
	R: GCTGTTAAGTTCTCCACGCC				
FV_tet_34	F: *AAAGAGGTTCATCAGGACAGAG	(AAAT)_8_	303–327	NED	MN259490
	R: GGTGAATCGCTACAGGTGTG				
FV_di_03	F: *CTCTGAGACGCCCAACTTC	(AG)_11_	100–140	FAM	MN259476
	R: AGGGTTCAGGACTTGTTGTAC				
FV_di_12	F: *GCAAGCCCTGTAAATGCAAC	(AG)_13_	171–203	VIC	MN259481
	R: CGTGTCTTTCTGTTGCCATTG				
FV_di_06	F: *ACTCGTGCCATCTCTCCTG	(AT)_19_	211–243	FAM	MN259477
	R: GGGTAGGCATGAAGAGCTAAAC				
FV_tri_27	F: *GAACCGAGCACTTTGAAGC	(AAG)_14_	183–216	PET	MN259488
	R: CCCTTCCCTGCCTTGAATTTC				
FV_tet_38	F: *GGAACGTACTAACCTCACCC	(AAAT)_6_	312–332	NED	MN259491
	R: GGCGATCTCTTCAGTTTGAGTC				
FV_di_07	F: *CGATCGAGCCTGTAAATGAC	(AG)_16_	231–270	FAM	MN259478
	R: AGGTTGAAGCTATCGTTGGG				
FV_di_47	F: *CCGACGATCCTAGCTCTTC	(AT)_15_	131–161	FAM	MN259480
	R: GATCGTGAAATGAGACCGGC				
FV_di_54	F: *ACCACCTCCACATCTTCGAG	(AC)_13_	243–275	VIC	MN259483
	R: AGATCGAGGCACCACAGATC				
FV_tri_58	F: *GGGCGATTGATTTCAGGAG	(ATC)_14_	189–225	PET	MN259485
	R: CCTGTAAACCATGGCGCTTC				
FV_tri_60	F: *GGGACTCTTATTGCCGTCG	(AAT)_18_	134–179	NED	MN259489
	R: CAAGATGCTGCACCAAATCC				
FV_tri_59	F: *CCTTTCAAACCCACGCATTG	(ATC)_17_	141–192	PET	MN259487
	R: TTGGACACATTTGGAGTCGC				
FV_di_53	F: *GCATTTAGACATCGCAAAGG	(AC)_22_	268–294	FAM	MN259482
	R: CTGCTGCCTTGGTGAGCC				
FV_tri_56	F: *AACCACCAAACCCTAAACCC	(AAC)_10_	212–239	VIC	MN259484
	R: TAGGTCAGGTGGTTGTTGGG				

^a^ Annealing temperature was 55°C for all loci. An asterisk (*) in front of a forward sequence represents the M13 tag (GGAAACAGCTATGACCAT).

### Microsatellite data analysis and results

Summary statistics were calculated for each microsatellite locus and population combination, i.e., the number of alleles, observed and expected heterozygosities, and inbreeding index *f* (Weir and Cockerham, 1984), as a measure of departure from within‐population random mating using the R package diveRsity (Keenan et al., 2013). Deviation from Hardy–Weinberg equilibrium was determined by the Fisher's exact test as implemented in diveRsity with 9999 Monte Carlo replicates. In order to reduce the number of false‐positive results, a Bonferroni correction was used. MICRO‐CHECKER version 2.2.3 (van Oosterhout et al., 2004), using the method of Chakraborty et al. (1994), was used to identify potential scoring errors due to stuttering, large allele dropouts, and the presence of null alleles in the matrix.

The summary statistics for genetic variability of the loci and populations studied are presented in Table 2. We identified a total of 203 alleles at 17 microsatellite loci, ranging from three to 15 per locus. Levels of observed heterozygosity ranged from 0.267 to 1.000, and levels of expected heterozygosity ranged from 0.461 to 0.899. Inbreeding coefficients of all three populations were relatively high (Table 2), indicating a certain level of inbreeding in each population, thus conflicting with the results of Wiedema et al. (2000) based on isoenzyme analyses. Accordingly, Hardy–Weinberg equilibrium tests indicated that eight out of 17 primer pairs deviated significantly from the expected values (Table 2) in one or two populations, which could be expected by small and inbred populations or presence of null alleles. The average null allele frequency for each locus calculated using the method of Chakraborty et al. (1994) detected a moderate presence of null alleles below 0.2. Five loci showed null allele frequencies over 0.2, but these were not present in all three populations (Table 2).

Cross‐amplification testing showed seven successfully cross‐amplified loci in *F. camtschatica* and *F. ulmaria*, and three successfully cross‐amplified loci in *Geum urbanum* (Table 3).

**Table 2:** Genetic parameters of the 17 polymorphic nuclear microsatellite loci developed for three populations of *Filipendula vulgaris*.^a^

Locus	pop1 (*n* = 20)						pop3 (*n* = 20)						pop56 (*n* = 20)					
	ch	*A*	*H*_o_	*H*_e_	*f*	HWE^b^	ch	*A*	*H*_o_	*H*_e_	*f*	HWE^b^	ch	*A*	*H*_o_	*H*_e_	*f*	HWE^b^
FV_di_02	0.070	6	0.650	0.748	0.130	ns	–0.054	6	0.875	0.785	–0.114	ns	0.044	8	0.765	0.836	0.109	ns
FV_di_10	0.108	8	0.611	0.759	0.195	***	0.107	7	0.471	0.583	0.193	***	0.122	7	0.563	0.719	0.085	ns
FV_tri_24	0.087	7	0.647	0.770	0.160	ns	–0.094	8	1.000	0.828	–0.207	ns	0.064	6	0.600	0.682	0.217	ns
FV_tet_34	–0.042	4	0.571	0.526	–0.087	ns	–0.114	5	0.632	0.503	–0.256	ns	0.234	5	0.438	0.705	0.121	ns
FV_di_03	0.122	6	0.563	0.719	0.217	ns	0.113	10	0.632	0.792	0.203	***	0.003	12	0.833	0.838	0.380	ns
FV_di_12	0.052	12	0.789	0.877	0.100	ns	–0.071	10	1.000	0.867	–0.153	ns	0.123	12	0.688	0.881	0.006	ns
FV_di_06	0.053	10	0.737	0.820	0.101	ns	0.151	11	0.650	0.881	0.262	***	0.082	9	0.706	0.832	0.220	*
FV_tri_27	–0.012	7	0.684	0.668	–0.025	ns	0.069	7	0.650	0.746	0.129	ns	0.037	11	0.824	0.888	0.152	ns
FV_tet_38	–0.052	3	0.579	0.522	–0.109	ns	0.015	3	0.556	0.573	0.030	ns	0.248	2	0.278	0.461	0.072	ns
FV_di_07	0.026	12	0.846	0.891	0.050	ns	0.059	10	0.722	0.813	0.112	ns	0.094	14	0.737	0.889	0.398	ns
FV_di_47	0.100	10	0.647	0.791	0.182	***	0.371	9	0.368	0.803	0.541	***	0.008	9	0.750	0.763	0.171	ns
FV_di_54	0.093	9	0.647	0.780	0.171	***	–0.004	8	0.800	0.794	–0.008	ns	0.063	8	0.688	0.779	0.016	ns
FV_tri_58	0.046	8	0.688	0.754	0.088	***	–0.035	7	0.789	0.737	–0.071	ns	–0.002	7	0.778	0.775	0.118	ns
FV_tri_60	0.064	9	0.714	0.811	0.119	ns	0.065	15	0.789	0.899	0.122	***	0.431	6	0.267	0.671	–0.004	***
FV_tri_59	0.058	10	0.737	0.828	0.110	***	0.022	8	0.789	0.824	0.042	ns	–0.029	10	0.846	0.799	0.603	ns
FV_di_53	0.154	12	0.632	0.861	0.267	ns	0.060	10	0.786	0.885	0.112	ns	0.126	10	0.667	0.860	–0.059	ns
FV_tri_56	0.250	6	0.400	0.666	0.400	ns	0.058	7	0.688	0.771	0.109	ns	0.007	4	0.467	0.473	0.224	ns
Overall		139	0.660	0.750	0.129	***		141	0.720	0.770	0.068	***		140	0.640	0.760	0.153	***

Note

*A* = number of alleles; ch = null allele frequency averaged over all populations using the method of Chakraborty et al. (1994); *f* = inbreeding coefficient; *H*_e_ = expected heterozygosity; *H*_o_ = observed heterozygosity; HWE = Hardy–Weinberg equilibrium; *n* = number of individuals sampled.

a Voucher and locality information are provided in Appendix 1.

b Deviation from HWE after Bonferroni correction (ns = not significant, *P < 0.05, **P < 0.01, ***P < 0.001).

**Table 3:** Results of cross‐amplification of 17 microsatellite markers developed for *Filipendula vulgaris* and tested in three species of the same family.^a^

Locus	*Filipendula camtschatica* (*n* = 5)	*Filipendula ulmaria* (*n* = 5)	*Geum urbanum* (*n* = 5)
FV_di_02	160–179	163–173	—
FV_di_10	—	—	—
FV_tri_24	—	—	—
FV_tet_34	—	—	325
FV_di_03	88–116	95–124	—
FV_di_12	163–175	157–167	142–178
FV_di_06	179–184	179–184	—
FV_tri_27	—	—	—
FV_tet_38	—	—	—
FV_di_07	244–275	244–261	—
FV_di_47	—	—	—
FV_di_54	—	—	—
FV_tri_58	159–168	159–168	—
FV_tri_60	—	—	—
FV_tri_59	—	—	135
FV_di_53	244–276	288–320	—
FV_tri_56	—	—	—

Note

*—* = unsuccessful amplification; *n* = number of individuals sampled.

^a^Voucher and locality information are provided in Appendix 1.

## Conclusions

Seventeen polymorphic microsatellite loci were successfully developed for *F. vulgaris*. The cross‐species amplification of these markers indicates that half of them may also be useful in the related species *F. camtschatica*,* F. ulmaria*, and *G. urbanum*. The markers developed here constitute a valuable tool for genetic investigation of population structure, gene flow levels, and mating systems, as well as conservation genetic studies of the *Filipendula* genus and will facilitate ecological and evolutionary studies of *F. vulgaris* and related species.

## Author contributions

D.Č., K.K., P.V., and B.M. conceived and designed the study. K.K., P.V., and B.M. collected the plant material. D.Č. and K.K. supervised the laboratory work. D.Č. and B.M. analyzed the data and drafted the manuscript. All authors reviewed the manuscript and approved its final version.

## Data Availability

All sequence information was deposited in the National Center for Biotechnology Information (NCBI) GenBank database (accession numbers MN259475–MN259491). The NCBI BioSample accession number for Illumina sequencing is SAMN12646161, as a part of BioProject no. PRJNA562628. Raw reads are stored in the NCBI Sequence Read Archive (SRR10028579).

## Appendix 1 Locality and voucher information for populations of *Filipendula vulgaris*,* F. camtschatica*,* F. ulmaria*, and *Geum urbanum*.

**Table:**

Taxon	Population code	Voucher no.^a^	*n*	Location	Geographic coordinates	Elevation (m)
*Filipendula vulgaris* Moench	pop1	BRNU 665928	20	Czech Republic, Louny	50.4102239°N, 13.8070417°E	476
*Filipendula vulgaris*	pop3	BRNU 667301	20	Poland, Raclawice, Waly	50.3382670°N, 20.2333170°E	300
*Filipendula vulgaris*	pop56	BRNU 667336	20	Bosnia and Herzegovina, Banja Luka	44.9034444°N, 17.3384444°E	120
*Filipendula camtschatica* (Pall.) Maxim.	—	BRNU 667333	5	Czech Republic, Stříbrná	50.3781700°N, 12.5473400°E	660
*Filipendula ulmaria* (L.) Maxim.	—	BRNU 667331	5	Czech Republic, Nový Bor	50.7824058°N, 14.5456506°E	470
*Geum urbanum* L.	—	BRNU 667279	5	Czech Republic, Prague–Suchdol	50.1350822°N, 14.3725494°E	300

Note

*n* = number of individuals sampled.

^a^ Herbarium vouchers are deposited at the herbarium of the Department of Botany and Zoology of Masaryk University, Brno (BRNU).
